# Lipidomic analysis of brain tissues and plasma in a mouse model expressing mutated human amyloid precursor protein/tau for Alzheimer’s disease

**DOI:** 10.1186/1476-511X-12-68

**Published:** 2013-05-09

**Authors:** Yoko Tajima, Masaki Ishikawa, Keiko Maekawa, Mayumi Murayama, Yuya Senoo, Tomoko Nishimaki-Mogami, Hiroki Nakanishi, Kazutaka Ikeda, Makoto Arita, Ryo Taguchi, Alato Okuno, Ryuta Mikawa, Shumpei Niida, Osamu Takikawa, Yoshiro Saito

**Affiliations:** 1 Project team for disease metabolomics, National Institute of Health Sciences, Tokyo, 158-8501, Japan; 2 Research Center for Biosignal, Akita University, Akita, 010-8543, Japan; 3 Institute for Advanced Biosciences, Keio University, Tsuruoka, Yamagata, 997-0052, Japan; 4 Departments of Health Chemistry, Graduate School of Pharmaceutical Sciences, University of Tokyo, Tokyo, 113-0033, Japan; 5 College of Life and Health Sciences, Chubu University, Kasugai, Aichi, 487-8501, Japan; 6 Research Institute, National Center for Geriatrics and Gerontology, Obu, Aichi, 474-8511, Japan; 7 Biobank, National Center for Geriatrics and Gerontology, Obu, Aichi, 474-8511, Japan; 8 Division of Medicinal Safety Science, National Institute of Health Sciences, 1-18-1 Kamiyoga, Setagaya-ku, Tokyo, 158-8501, Japan

**Keywords:** Lipidomic analysis, Alzheimer’s disease, Mice model, Ethanolamine plasmalogens, Oxidative fatty acids

## Abstract

**Background:**

Alzheimer’s disease (AD), the most common cause of dementia among neurodegenerative diseases, afflicts millions of elderly people worldwide. In addition to amyloid-beta (Aβ) peptide and phosphorylated tau, lipid dysregulation is suggested to participate in AD pathogenesis. However, alterations in individual lipid species and their role in AD disease progression remain unclear.

**Methods:**

We performed a lipidomic analysis using brain tissues and plasma obtained from mice expressing mutated human amyloid precursor protein (APP) and tau protein (Tg2576×JNPL3) (APP/tau mice) at 4 (pre-symptomatic phase), 10 (early symptomatic) and 15 months (late symptomatic).

**Results:**

Levels of docosahexaenoyl (22:6) cholesterol ester (ChE) were markedly increased in APP/tau mice compared to controls at all stages examined. Several species of ethanolamine plasmalogens (pPEs) and sphingomyelins (SMs) showed different levels between brains from APP/tau and control mice at various stages of AD. Increased levels of 12-hydroxyeicosatetraenoic acid (12-HETE) during the early symptomatic phase were consistent with previous reports using human AD brain tissue. In addition, 19,20-dihydroxy-docosapentaenoic acid (19,20-diHDoPE) and 17,18-dihydroxy-eicosatetraenoic acid (17,18-diHETE), which are produced from docosahexaenoic acid and eicosapentaenoic acid via 19,20-epoxy-docosapentaenoic acid (19,20-EpDPE) and 17,18-epoxy-eicosatetraenoic acid (17,18-EpETE), respectively, were significantly increased in APP/tau brains during the pre-symptomatic phase, and concomitant increases occurred in plasma. Several arachidonic acid metabolites such as prostaglandin D_2_ (PGD_2_) and 15-hydroxyeicosatetraenoic acid (15-HETE), which have potential deteriorating and protective actions, respectively, were decreased in the early symptomatic phase of APP/tau mice. Significant decreases in phosphatidylcholines and PEs with polyunsaturated fatty acids were also detected in the late symptomatic phase, indicating a perturbation of membrane properties.

**Conclusion:**

Our results provide fundamental information on lipid dysregulation during various stages of human AD.

## Background

Alzheimer’s disease (AD) afflicts millions of elderly people worldwide and is the most common cause of dementia among neurodegenerative diseases. AD symptoms include impaired memory, aphasia, and visuospatial deficits while the histopathological changes of AD are characterized by senile plaques, neurofibrillary tangles, and lipid granule accumulation. The major molecular component of senile plaques is aggregated amyloid-β (Aβ) peptide, which is generated from the amyloid precursor protein (APP) via sequential cleavage by β-secretase and the γ-secretase complex [1]. AD onset is assumed to be related to Aβ peptide polymerization. On the other hand, neurofibrillary tangles are composed of aberrantly phosphorylated tau protein [2].

In addition to the above pathological features, numerous studies reported a link between lipids and AD. In general, membrane lipids provide a milieu for transmembrane proteins and can modulate their function. Indeed, γ-secretase activity is affected by the lipid composition of the membrane with sphingolipids and cholesterol increasing and phosphatidylinositols decreasing its activity [3,4]. Lipid carbon chain length and double bond position also affect γ-secretase activity. Hypercholesterolemia reportedly accelerates intraneuronal accumulation of Aβ oligomers and subsequent synapse loss, resulting in memory impairment in AD model mice [5]. Moreover, ω-3 polyunsaturated fatty acids were shown to have possible roles in AD prevention [6]. The docosahexaenoic acid (DHA) content of phospholipids (PLs) was lower in brain tissue and plasma of AD patients compared to those without cognitive impairment [7]. In addition, neuroprotectin D1, which is derived from DHA, was related to suppression of Aβ_42_-induced neurotoxicity [8]. Several lipidomic analyses for AD were recently reported. For instance, an analysis of postmortem brain tissues obtained from patients with late-onset AD showed enrichment in lysobisphosphatidic acids, sphingomyelins (SMs), ganglioside GM3, and cholesterol esters (ChEs) as well as novel region-specific lipid anomalies that were potentially linked to AD pathogenesis [9]. However, our current knowledge of the overall changes in lipids related to AD, especially for the time-course of their changes, remains low.

Animal models are extensively used to study AD pathogenesis since brain samples can be obtained from pre-symptomatic to late stages of AD, which allows analysis of pathophysiological changes at different disease stages. A recent study showed that some lipid dysregulation occurred in postmortem AD brain tissues, including increased levels of ganglioside GM3 and ChEs, which was consistent with changes seen in AD mouse models [9], and supports the validity of using animal models to study AD disease mechanisms. In this study, we performed a global lipid metabolomic analysis using brain tissues and plasma obtained from transgenic mice expressing both mutated human APP and tau proteins [10] at 4 months (pre-symptomatic), 10 months (early disease stage), and 15 months (late stage). We focused not only on lipid changes observed in the brain but also on those that were consistently changed in both brain and plasma because these molecules could be candidate biomarkers for AD diagnosis.

## Materials and methods

### Animals

We used female transgenic mice hemizygous for transgenes encoding both human mutated APP and tau (APP/tau mice) as AD model mice. The mice were generated by crossing Tg2576 male transgenic mice hemizygously expressing human APP containing Swedish type mutations (Lys670Asn, Met671Leu) with JNPL3 female transgenic mice homozygously expressing mutant P301L four-repeat tau proteins. Wild-type tau females crossed with wild-type APP male mice were used as a control (no transgenes). Two-month-old mice were purchased from Taconics Farms, Inc. (Hudson, NY, USA) and raised to 4 (pre-symptomatic), 10 (early stage of symptoms), and 15 months (late stage) of age. At the given time point, mice were anesthetized with 2.0-2.5% isoflurane in the atmosphere and blood from the inferior vena cava was collected in tubes containing EDTA-2Na for plasma preparation. After blood collection, mouse brains were excised, and the cerebral hemispheres without the cerebellum were weighed and frozen in liquid nitrogen before storage at −80°C. Each group consisted of 5 mice except for the 15-month-old APP/tau mice (N=3). All animal experiments were performed in accordance with the Guidelines for Animal Experimentation and with approval from the Ethics Committee of Animal Care and Experimentation of the National Center for Geriatrics and Gerontology, Japan.

### Assay of human Aβ_40_ and tau proteins

The right cerebral hemisphere from each animal (*ca*. 150 mg) was homogenized as described below in the section describing lipid extraction. After removal of methanol with a centrifugal vacuum concentrator (TOMY), Aβ_40_ in brain tissues (2 mg) was extracted by homogenization in 0.2 ml 70% formic acid with a small Dounce homogenizer and centrifugation at 100,000 × g for 1 h at 4°C in an ultracentrifuge (Optima MAX-E, Beckman Coulter, Tokyo, Japan). The supernatants were recovered and stored at −80°C until analysis. Frozen formic acid extracts were thawed on ice and neutralized with 20 volumes of 1 M Tris base. Within 1 h of neutralization, Aβ_40_ levels in the extracts were determined by an Enzyme-Linked Immunosorbent Assay (ELISA) kit for human Aβ_40_ (Wako Pure Chemical Industries, Ltd., Osaka, Japan) according to the manufacturer’s instructions. Total protein in the extracts was assayed by a Pierce® BCA™ Protein Assay Kit (Thermo Fisher Scientific Ltd., Yokohama, Japan).

Human tau protein in brain tissue from each mouse was analyzed according to the method described by Lewis et al. [10]. Two mg of EtOH-free homogenized brain tissue from each mouse was homogenized with a small Dounce homogenizer in 0.2 ml of tris-buffered saline (TBS) and centrifuged at 100,000 × g for 1 h at 4°C. The supernatant was collected as the TBS-soluble fraction. The pellet was homogenized similarly in 0.2 ml of 0.8 M NaCl and 10% sucrose in TBS, and after centrifugation at 150,000 × g for 15 min at 4°C, the supernatant was brought to 1% sarkosyl and incubated at 37°C for 1 h. The mixture was then centrifuged at 150,000 × g for 30 min at 4°C whereupon the supernatant and precipitate were collected as the sarkosyl-soluble and -insoluble fraction, respectively. TBS-soluble and sarkosyl-soluble fractions were combined and defined as the soluble fraction. Human tau levels in the soluble and sarkosyl-insoluble fractions were determined using an ELISA kit for human tau (Lifetechnology Japan Ltd., Tokyo, Japan). The sarkosyl-insoluble fraction was dissolved in 0.1 ml of 70% formic acid and neutralized with 20 volumes of 1M Tris base immediately before ELISA determination.

### Lipid extraction

Total lipids from right cerebral hemisphere tissues or plasma were extracted using Bligh & Dyer’s (BD) method with minor modifications [11]. The right cerebral hemisphere from each animal (*ca*. 150 mg) was homogenized with zirconia beads in 1 mL of methanol at 4°C using a cell disruptor (TOMY), and the equivalent of 10 mg tissue was transferred into glass tubes. Plasma (100 μl) diluted with methanol was also transferred into glass tubes. A mixture of internal standards (ISs) was added to tissue homogenates or plasma: 1, 2-dipalmitoyl D6-3-*sn* glycerophosphatidylcholine (16:0/16:0PC-d6, 40 nmol/10 mg tissue or 20 nmol/100 μl plasma, Larodan, Malmo, Sweden), 1,2-caprylin-3-linolein (250 pmol and 20 nmol, Larodan), ^13^C-labelled tripalmitin (tripalmitin-1,1,1-^13^C_3_, 250 pmol and 2 nmol, Larodan), deuterated prostaglandin E_2_ (PGE_2_-d4, 5 ng in both tissue and plasma, Cayman Chemical, Ann Arbor, MI, USA) and deuterated leukotriene B_4_ (leukotriene B_4_-d4, 5 ng in both tissue and plasma, Cayman Chemical). Then, chloroform, methanol and 20 mM potassium phosphate (Kpi) buffer were added to achieve a volume ratio of buffer/methanol/chloroform = 0.8/2/1, and mixed vigorously for 5 min. Phase separation was achieved by adding 1 ml each of chloroform and 20 mM Kpi buffer and vortexing. The mixture was centrifuged at 1,000 × g for 10 min. For brain tissue, both the upper aqueous layer and bottom organic layer were collected. For plasma, 100 mM KCl/methanol/chloroform (48/47/3) was further added to the remaining organic samples after the upper aqueous layer was collected. After mixing vigorously for 5 min and centrifugation at 1,000 x g for 10 min, only the bottom organic layer was collected. Samples of the organic layer were dried under a gentle stream of nitrogen, and then dissolved in chloroform-methanol (1:1) at a concentration of 10 mg (tissue weight)/ml or 100 μL (plasma volume)/ml, and were stored at −90°C until use (BD sample). To distinguish alkenylacyl and alkyl PL species with the same exact mass as 34:1 ethanolamine plasmalogen (pPE) and 34:2 alkylacyl PE, a small aliquot of each BD sample was hydrolyzed using 0.5 N HCl as described previously (BD acid hydrolysis sample) [12].

Samples of the aqueous layer (3.2 ml) were subjected to solid extraction to obtain oxidative fatty acids. First, samples were diluted 10-fold using water adjusted to pH 3.0 with 1N HCl, and then applied to Oasis SPE cartridges (60 mg, Waters, Millford, MA, USA) preconditioned with 3 ml each of methanol and Milli Q water. After columns were washed with 3 ml of Milli-Q water followed by 3 ml hexane, the oxidative fatty acids were eluted with 3 ml methyl formate (MF). The MF fraction was dried under nitrogen, dissolved in chloroform-methanol (1:1) at a concentration of 10 mg (tissue weight)/ml or 100 μL (plasma volume)/ml, and stored at −90°C until use.

### Lipid metabolite analysis by reverse-phase liquid chromatography (RPLC)-time of flight mass spectrometry (TOFMS)

Total lipid samples (BD samples and BD acid hydrolysis samples) were analyzed by electrospray ionization (ESI)-TOFMS (LCT Premier XE; Waters Micro-mass, Waters) interfaced with an Acquity UPLC System (Waters). The MS was operated in a W-optics mode with 10,000 and 8,000 resolution in the positive and negative modes, respectively, using dynamic range extension. The scan range of the instrument was set at a mass-to-charge ratio (*m*/*z*) of 150–1200, and three functions (positive and negative ion modes, and another negative ion mode for in-source fragmentation) were recorded simultaneously for lipid species identification and quantification. The capillary voltage in the positive ion mode was set at 3.0 kV and the cone voltage 40 V, whereas in the negative ion mode the capillary and cone voltage was 2.5 kV and -40V, respectively. In in-source fragmentation of the negative ion mode, the aperture 1 voltage was set at 60 V. The desolvation gas was set to 600 L h^−1^ at a temperature of 350°C; the cone gas was set to 50 L/h and the source temperature was 120°C. The data acquisition rate was 0.5 s with a 0.01 s interscan delay. All analyses were acquired using the lock spray to ensure accuracy and reproducibility. A lock-mass of leucine enkephalin at 0.6 ng/mL in 50:50 acetonitrile:water containing 0.1% formic acid was used for the positive and negative ion modes ([M+H]^+^ = 556.2771 and [M+H]^-^ = 554.2615) with a flow rate of 5.0 μL/min via a lock spray ionization source. The data were collected in the centroid mode using MassLynx (Waters).

Before the RPLC-ESI-TOFMS analysis, the dried BD and BD acid hydrolysis samples were redissolved in chloroform/methanol/isopropanol (1:2:4) supplemented with 0.2% formic acid and 0.028% ammonia at a final concentration of 10 mg tissue/ml solution or 100 μl plasma/ml solution, and filtered through a 0.2 μm pore size polyvinylidene difluoride membrane filter (Millipore, Bedford, MA, USA). The injection volume was fixed at 2 μL, and an ACQUITY UPLC BEH C18 column (1.7 mm i.d. × 150 mm, 1.7 μm) was used for separation. The column temperature was maintained at 45°C. The flow rate of the mobile phase was 70 μL/min. Mobile phase A consisted of acetonitrile/methanol/water = 18/18/4 (0.1% formic acid and 0.028% ammonia), while mobile phase B consisted of isopropanol (0.1% formic acid and 0.028% ammonia). The linear gradient increased from 0 to 15% B over 5 min, from 15% to 30% B for the next 20 min, from 30% to 45% B during the next 15 min, from 45% to 60% B for the next 10 min, from 60% to 80% B for the next 5 min, and immediately ramped to 85% B for the final 12 min. Blank runs were carried out randomly between samples to confirm that there was no chromatographic carryover.

### Structural analysis of lipid metabolites by RPLC-ESI-linear ion trap-MS

For structural analysis of PL, SM and triacylglycerol (TAGs) species, BD samples were also analyzed using a Finnigan LTQ linear ion trap mass spectrometer (Thermo Fisher Scientific Inc. San Jose, CA, USA) interfaced with a Shimadzu Prominence HPLC system (Shimadzu, Kyoto, Japan). To analyze the fatty acid composition of PLs and SMs, data-dependent MS/MS (MS2) or MS3 analysis was performed in negative ion mode with an ion spray voltage of 4 kV and a scan range of *m*/*z* = 400–1000 as described previously [12]. For structural analysis of TAGs, data-dependent MS2 analysis was performed in the positive ion mode with an ion spray voltage of 4.5 kV and a scan range of *m*/*z* = 300–2000. The trap fill-time was 50 ms (full mass analysis) and 100 ms (MS^n^ analysis) in both ion modes. Nitrogen was used as a sheath gas (25 arbitrary units), and helium as a collision gas at a collision energy setting of 35%. BD samples were treated in the same way as RPLC-ESI-TOFMS analysis and applied onto an ACQUITY UPLC BEH C18 column (1.7 mm i.d. × 100 mm, 1.7 μm, Waters) kept at 45°C. LC conditions were the same as those in RPLC-ESI-TOFMS analysis. The classes of individual lipid species and their fatty acid compositions were determined with these data, together with RPLC-ESI-TOFMS data with exact mass and in-source fragmentation information.

### Targeted analysis of oxidative fatty acids by RPLC-ESI-triple quadrupole MS/MS

Oxidative fatty acids were measured as reported previously [13]. Briefly, MF fractions in solid extraction were analyzed by a 5500QTRAP quadrupole-linear ion trap hybrid mass spectrometer (AB Sciex, Framingham, MA, USA) interfaced with an ACQUITY UPLC System (Waters) equipped with an ACQUITY BEH C18 column (1.7 mm i.d. × 150 mm, 1.7 μm, waters), kept at 40°C. Samples were eluted with a mobile phase of water/acetate (100:0.1, v/v, solvent A’) and acetonitrile/methanol (4:1, v/v, solvent B’). The gradient increased from 27 to 50% B’ in five min, from 50 to 80% B’ in the next 30 min, from 80 to 100% B’ in the next five min, and kept at 100% B’ for ten min with flow rates of 50 μL/min (0 – 35 min), 50–100 μL/min (35 – 40 min), and 100 μL/min (40 – 50min). Analysis was performed with multiple reaction monitoring in the negative ion mode, with ion spray voltage, -4500 V; curtain (nitrogen), 10 arbitrary units; collision gas (nitrogen), 6 arbitrary units and a gas temperature of 350°C. We simultaneously measured 31 arachidonic acid metabolites, 14 eicosapentaenoic acid (EPA) metabolites, 13 DHA metabolites, 2 docosapentaenoic acid metabolites, and 2 linolenic acid metabolites. Declustering potential, collision energy and collision cell exit potential were optimized for each individual metabolite using authentic standard compounds (Cayman Chemical). In plasma samples from 15 month old control mice, one sample was omitted from the measurement due to instrumental problems (N=4).

### Data processing

RPLC-ESI-TOFMS data were subsequently processed using the 2DICAL software package (2 Dimensional Image Converted Analysis of LCMS, Mitsui Knowledge Industry Co. Ltd., Tokyo, Japan) [14]. This software allowed detection and alignment of the ion-peak intensity derived from biomolecules identified with *m*/*z* and column retention times (RTs) in an amount of collected data. The main parameters of 2DICAL were set as follows: RT range 0.1–60.0 min and 0–37.5 min in the positive and negative ion modes, respectively, a mass range 400 to 1200 *m*/*z*, mass tolerance 0.3 *m*/*z*, mass window 0.3 *m*/*z*, an RT window of 0.60 min, and noise elimination level 2. The resulting three-dimensional matrix contains an arbitrarily assigned peak index (RT-*m*/*z* pairs) and ion intensity information (variables). The extracted ion peaks of each metabolite were normalized to that of the ISs. 16:0/16:0PC-d6 (Larodan Fine Chemicals) was used as an IS to normalize metabolites of PLs, lysoPLs, SMs, ceramides (Cers) and diacylglycerols (DAGs), which eluted at 0.1 to 37.5 min in RPLC. To normalize the TAG and ChE metabolites that eluted at 37.5 to 60 min, Tripalmitin-1,1,1-^13^C_3_ (Larodan Fine Chemicals) was used.

Data for oxidative fatty acids were subsequently processed with Analyst™ (ver. 1.5.1, AB Sciex) software or MultiQuant™ Software (Version 2.1, AB Sciex). The integrated peak area of each metabolite was normalized to that of the IS (leukotriene B_4_-d4).

### Statistical and multiple classification analysis

The IS-normalized peak list from RPLC-TOFMS analysis was first exported to the SIMCA-P+ for multivariate data analysis (Infocom Corp., Tokyo, Japan). Orthogonal partial least-squares-discriminant analysis (OPLS-DA) was used to select variables that strongly contributed to the difference between the two mice groups of the same ages. The Welch’s *t*-test was then applied to assess statistical measures between the APP/tau and wild-type mice. For oxidative fatty acid analysis, Welch’s *t*-tests were directly applied to the results with normalized peak areas.

## Results

### Expression levels of Aβ_40_ and tau proteins in APP/tau mice

We measured the protein expression levels of Aβ_40_ and tau in APP/tau mice by ELISA. In APP/tau mice, Aβ_40_ accumulated in the brain from 10 months of age (Additional file 1: Figure S1). On the other hand, soluble human tau protein was expressed at similar levels from 4 through 15 months of age. The levels of sarkosyl-insoluble tau appeared to be slightly higher at 10 and 15 months than at 4 months, but these differences did not reach statistical significance among all ages tested. In wild-type mice, both Aβ_40_ and human tau were under the detection limit at all tested ages.

### Analysis of brain tissues

We first performed lipidomic analysis of BD samples extracted from brain tissues of APP/tau and wild-type mice at 4, 10, and 15 months of age using RPLC-ESI-TOFMS. When creating a two-dimensional map of RT versus *m/z* values of the individual precursor ion peaks in the positive ion mode, the intensities of a few spots that were apparently identified as ChEs were higher in APP/tau mice compared to wild-type mice at 4 months (Additional file 2: Figure S2). Likewise, at 10 and 15 months, these ChE spots retained the higher intensities in APP/tau mice compared to age-matched wild-type mice (data not shown). In the positive ion mode, a total of 803 peaks were detected and divided into two groups based on their retention time (RT). For RTs of 0.1–37.5 min, the 695 peaks contained mainly glycerophospholipids such as PLs, lysoPLs and sphingolipids such as SMs and Cers. For RTs of 37.5–60 min, the 108 peaks represented neutral lipids such as TAGs and ChEs. Peaks in each RT category were then separately processed by OPLS-DA (Additional file 3: Figure S3) because glycerophospholipids and neutral lipids are the two major classes of lipid metabolites with different physical properties. The OPLS-DA score plots for brain tissues indicated that APP/tau and wild-type mice could be reliably discriminated using established models at 4 (Additional file 3: Figure S3), 10 and 15 months (data not shown).

**Figure 1:**
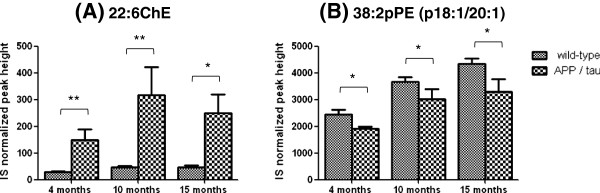
**Changes in 22:6ChE and 38:2pPE (p18:1/20:1) levels in brain tissue from APP/tau and wild-type mice.** The relative levels of metabolites are represented by the IS normalized peak heights using 16:0/16:0PC-d6 for 38:2pPE and tripalmitin-1,1,1-13C3 for 22:6ChE as ISs. These two metabolites were significantly changed in the brain across the tested ages. *p< 0.05, **p< 0.01 by Welch’s *t*-test. Values are mean ± SD of N = 3–5.

To detect with high confidence the metabolites contributing to the discrimination between the two groups (APP/tau vs. wild-type) that were located at extreme ends of “S” of the OPLS-DA loading S-plot, we screened variables from this plot with threshold values of w[1] > |0.05|, p(corr)[1] > |0.6|, which represent the magnitude of contribution (weight) and reliability (correlation), respectively. For brain tissue from 4-month-old animals (pre-symptomatic phase), 9 and 12 metabolites in total (RT, 0.1-60 min) that exceeded this threshold value showed higher and lower levels in APP/tau compared to wild-type mice, respectively (Additional file 3: Figure S3). In addition, 8 and 8 metabolites at 10 months (early disease stage) and 5 and 58 metabolites at 15 months (late stage) had higher and lower levels in APP/tau compared to wild-type mice, respectively, and met the criteria (data not shown). The relative quantification data of the identified metabolites that meet the OPLS-DA loading S-plot criteria (unidentified peaks were omitted) were summarized in Additional file 4: Table S1. Of these metabolites, the levels of 2, 3 and 2 species were significantly increased and 4, 3 and 24 were significantly decreased in APP/tau mice compared to wild-type mice at 4, 10 and 15 months, respectively, by Welch’s *t*-test (Table 1). Particularly, docosahexaenoyl-ChE (22:6 ChE) and 38:2 pPE (p18:1/20:1) were consistently increased and decreased, respectively, across the tested ages in APP/tau mice compared to wild-type mice (Figure 1). Other ChE species such as 18:1ChE were under the detection limit (data not shown). Notably, many pPE species (e.g., 36:2pPE (p18:1/18:1), 36:4pPE (p16:0/20:4), 38:4pPE (p16:0/22:4), 38:4pPE (p18:0/20:4), 40:4pPE (p18:0/22:4), 40:6pPE (p18:0/22:6)) and SM species (e.g., 36:2SM (d18:2/N18:0), 38:1SM (d18:1/N20:0), 34:1SM (d18:1/N16:0)) were decreased at 15 months (Table 1). Decreases in several phosphatidylcholines (PCs) and PEs mainly with polyunsatulated fatty acids (PUFAs) were also detected in the late symptomatic phase (Table 1). As for triacylglycerol (TAG), the level of 60:12TAG (16:0/22:6/22:6) was increased (1.33-fold) in 10 month old animals, whereas most TAG levels were decreased in APP/tau mice compared to wild type mice at 15 months, and showed statistical significance for 52:3TAG (16:0/18:1/18:2, 16:1/18:1/18:1), 53:6TAG and 54:4TAG (16:0/18:0/20:4) (0.40, 0.54 and 0.31-fold, respectively) (Table 1 and Additional file 4: Table S1).

**Table 1:** Fold changes in significantly changed lipids in brain tissues

**Molecular species**	**4 months**		**10 months**		**15 months**	
	**Fold Change **^**1**^	***p*-value **^**2**^	**Fold Change **^**1**^	***p*-value **^**2**^	**Fold Change **^**1**^	***p*-value **^**2**^
***Phosphatidylcholine (PC)***						
30:0PC (14:0/16:0)	1.35	0.026*	1.14	0.200	1.03	0.198
32:0PC (16:0/16:0)	1.02	0.815	0.87	0.093	0.83	0.011*
34:2PC (16:0/18:2)	1.32	0.190	1.63	0.014*	1.03	0.282
36:3PC (16:0/20:3)	1.06	0.550	1.07	0.441	1.28	0.029*
38:1PC (18:0/20:1)	0.84	0.092	0.87	0.155	0.77	0.010**
38:4PC (16:0/22:4)	1.07	0.462	0.92	0.279	0.86	0.011*
38:5PC (18:1/20:4)	1.10	0.104	0.93	0.426	0.88	0.021*
40:4PC (18:0/22:4)	1.05	0.596	0.91	0.188	0.84	0.003**
40:7PC (18:1/22:6)	1.00	0.984	0.90	0.615	0.87	0.021*
34:1ePC	1.04	0.542	0.92	0.338	0.86	0.029*
***Phosphatidylethanolamine (PE)***						
38:1PE (20:0/18:1)	0.74	0.021*	0.89	0.413	0.66	0.024*
38:5PE (18:1/20:4)	0.99	0.942	0.94	0.352	0.85	0.023*
40:4PE (18:0/22:4)	1.10	0.265	0.91	0.269	0.89	0.035*
***Ethanolamine plasmalogen (pPE)***						
36:2pPE (p18:1/18:1)	0.76	0.299	0.88	0.183	0.86	0.042*
36:4pPE (p16:0/20:4)	1.02	0.810	0.91	0.359	0.89	0.027*
38:2pPE (p18:1/20:1)	0.78	0.037*	0.83	0.037*	0.76	0.034*
38:4pPE (p16:0/22:4)	1.02	0.682	0.92	0.310	0.82	0.009**
38:4pPE (p18:0/20:4)	0.94	0.819	0.93	0.796	0.87	0.029*
40:4pPE (p18:0/22:4)	0.96	0.611	0.94	0.399	0.88	0.013*
40:6pPE (p18:0/22:6)	1.02	0.822	0.89	0.182	0.87	0.043*
***Sphingomyelin (SM)***						
34:1SM (d18:1/N16:0)	0.98	0.863	0.97	0.675	0.76	0.007**
36:2SM (d18:2/N18:0)	0.94	0.335	0.81	0.010*	0.86	0.027*
38:1SM (d18:1/N20:0)	0.90	0.278	0.85	0.067	0.79	0.006**
42:2SM (d18:1/N24:1)	0.78	0.048*	0.86	0.388	0.60	0.066
***Cerebroside***						
42:2cerebroside	0.81	0.045*	0.92	0.334	0.84	0.137
***Diacylglycerol (DAG)***						
38:4DAG	0.94	0.420	0.74	0.016*	0.78	0.043*
***Triacylglycerol (TAG)***						
52:3TAG (16:0/18:1/18:2, 16:1/18:1/18:1)	0.87	0.353	0.68	0.143	0.40	0.002**
53:6TAG	1.05	0.765	0.91	0.648	0.54	0.003**
54:4TAG (16:0/18:0/20:4)	1.09	0.463	1.04	0.801	0.31	<0.001***
60:12TAG (16:0/22:6/22:6)	0.99	0.935	1.33	0.011*	0.87	0.544
***Cholesterol ester (ChE)***						
22:6ChE	5.22	0.002**	6.96	0.004**	5.43	0.032*

^1^ Fold change (ratio of APP/tau to wild-type) is the mean of N=3-5.

^2^ Welch’s *t*-test (*p<0.05, **p<0.01,***p<0.001).

**Figure 2:**
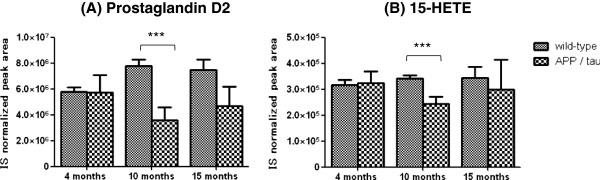
**Changes of prostaglandin D**_**2 **_**and 15-HETE levels in brain tissue from APP/tau and wild-type mice.** The relative levels of metabolites are represented by the IS normalized peak areas using leukotriene B_4_-d4 as an IS. ***p< 0.001 by Welch’s *t*-test. Values are mean ± SD of N = 3–5.

Free PUFAs and their oxidized metabolites extracted from brain tissues were then measured using a multiple reaction monitoring system, and 16 species were detected (Additional file 5: Table S2). Of these, 9 species were significantly changed between APP/tau and wild-type mice as shown in Table 2. The numbers of variable metabolites were 2, 7 and 0 species at 4, 10 and 15 months, respectively. No free PUFAs or their oxidized metabolites were consistently changed between APP/tau and wild-type mice across the ages tested (Additional file 5: Table S2). Pathway analysis of arachidonic acid metabolites is shown in Additional file 6: Figure S4. A marked decrease in prostaglandin D_2_ and 15-hydroxyeicosatetraenoic acid (HETE) was observed at 10 months (Figure 2).

**Table 2:** Fold changes of the significantly changed oxidized fatty acids in brain tissues

**Molecular species**	**4 months**		**10 months**		**15 months**	
	**Fold Changes **^**1**^	***p*-value **^**2**^	**Fold Changes **^**1**^	***p*-value **^**2**^	**Fold Changes **^**1**^	***p*-value **^**2**^
***Metabolites derived from arachidonic acid***						
Prostaglandin D_2_	0.99	0.941	0.46	< 0.001***	0.63	0.059
Thromboxane B_2_	1.24	0.113	0.66	0.013*	1.07	0.845
12-HHT	1.19	0.149	0.67	0.005**	0.98	0.952
12-HETE	0.90	0.777	1.77	0.049*	1.03	0.931
15-HETE	1.03	0.747	0.71	<0.001***	0.87	0.604
11,12-EpETrE	0.84	0.225	0.83	0.007**	1.04	0.886
14,15-EpETrE	0.85	0.053	0.79	0.029*	0.97	0.810
***Metabolites derived from eicosapentaenoic acid***						
17,18-diHETE	2.12	< 0.001***	1.10	0.602	1.42	0.300
***Metabolites derived from docosahexaenoic acid***						
19,20-diHDoPE	1.72	0.002**	1.20	0.251	1.38	0.261

^1^ Fold changes (ratio of APP/tau to wild-type) are means of N=3-5.

^2^ Welch’s *t*-test (*p<0.05, **p<0.01,***p<0.001).

HHT, hydroxy-heptadecatrienoic acid; HETE, hydroxyeicosatetraenoic acid; EpETrE, epoxyeicosatrienoic acid; diHETE, dihydroxyeicosatetraenoic acid; diHDoPE, dihydroxydocosapentaenoic acid.

### Analysis of plasma

In the positive ion mode of BD samples from plasma, 385 and 308 peaks were detected on the basis of their RT, 0.1-37.5 min and 37.5-60 min, respectively. Next, we analyzed changes in lipid species levels in plasma by OPLS-DA loading S-plot (data not shown) in the same manner as for the brain tissues. At 4 months of age, 35 and 3 metabolites (RT, 0.1-60 min) with higher and lower levels in APP/tau mice than in wild-type mice, respectively, were detected. Forty-five and 9 metabolites displayed higher and lower levels in APP/tau mice compared to wild-type mice, respectively, at 10 months, and 32 and 22 were detected at 15 months. Relative quantification results of these metabolites are shown in Additional file 7: Table S3 (unidentified peaks were omitted) and the fold changes of metabolites with significantly different levels between APP/tau and wild-type mice are listed in Table 3. The levels of 23 metabolites (22 were increased and 1 was decreased in APP/tau mice) at 4 months, 32 metabolites (26 and 6) at 10 months and 12 metabolites (7 and 5) at 15 months were significantly different between APP/tau and wild-type mice. As for PUFAs and their oxidized metabolites, the 34 species were detected (Additional file 8: Table S4) and, 7, 3 and 9 species levels were also significantly changed between the two mice groups at 4, 10, and 15 months, respectively (Table 4).

**Table 3:** Fold changes of the significantly changed lipids in plasma

**Molecular species**	**4 months**		**10 months**		**15 months**	
	**Fold Changes **^**1**^	***p*-value **^**2**^	**Fold Changes **^**1**^	***p*-value **^**2**^	**Fold Changes **^**1**^	***p*-value **^**2**^
***Phosphatidylcholine (PC)***						
16:1LPC	0.99	0.960	0.59	0.026*	1.07	0.724
18:0LPC	1.50	0.019*	1.27	0.281	1.01	0.921
20:5LPC ^3^	1.45	0.011*	0.82	0.440	1.26	0.190
22:6LPC	1.45	0.011*	1.03	0.889	1.10	0.619
34:1PC (16:0/18:1)	1.24	0.037*	1.00	0.983	1.27	0.071
34:2PC (16:0/18:2)	1.27	0.053	1.17	0.007**	1.21	0.015*
34:3PC (16:0/18:3)	1.06	0.698	0.65	0.034*	0.98	0.936
35:2PC	1.68	0.030*	1.55	0.013*	1.27	0.035*
36:1PC (18:0/18:1)	1.55	0.010**	1.16	0.256	1.19	0.109
36:2PC (18:0/18:2)	1.48	0.024*	1.35	0.017*	1.11	0.139
36:5PC (16:0/20:5)	1.51	0.011*	0.81	0.509	1.40	0.374
37:2PC (19:0/18:2)	1.33	0.144	1.47	0.008**	1.26	0.071
38:3PC (18:0/20:3)	1.52	0.045*	1.38	0.209	1.17	0.497
38:4PC (18:1/20:3)	0.99	0.957	0.63	0.031*	0.82	0.137
38:5PC (18:0/20:5)	1.74	0.008**	1.08	0.783	1.24	0.266
38:5PC (18:1/20:4)	1.13	0.331	0.79	0.018*	0.84	0.149
38:7PC	1.08	0.644	0.48	0.019*	0.94	0.807
40:4PC (20:0/20:4)	1.50	0.018*	1.47	0.077	1.12	0.313
40:5PC (18:0/22:5)	1.71	0.007**	1.25	0.182	1.30	0.349
40:6PC (18:0/22:6)	1.64	0.033*	1.39	0.099	1.06	0.629
40:7PC (18:1/22:6)	1.06	0.642	0.72	0.022*	0.73	0.072
42:6PC (20:0/22:6)	1.41	0.002**	1.52	0.012*	1.38	0.110
34:2ePC (16:0e/18:2)	1.52	<0.001***	1.81	<0.001***	1.47	0.011*
***phosphatidylinositol (PI)***						
38:5PI (18:0/20:5)	1.53	0.024*	0.92	0.748	2.00	0.002**
***Sphingomyelin (SM)***						
34:1SM (d18:1/N16:0)	1.25	0.057	1.35	0.027*	1.30	0.032*
42:2SM	1.37	0.051	1.39	0.024*	1.38	0.069
42:3SM (d18:2/N24:1)	1.48	0.030*	1.22	0.061	1.30	0.141
***Triacylglycerol (TAG)***						
52:3TAG (16:0/18:1/18:2)	1.21	0.557	1.21	0.558	0.58	0.045*
52:4TAG (16:0/18:2/18:2)	1.57	0.250	1.41	0.421	0.40	0.032*
52:5TAG (16:0/18:2/18:3)	1.04	0.903	0.74	0.314	0.49	0.011*
54:3TAG (18:0/18:1/18:2)	0.64	0.553	0.62	0.411	0.48	0.043*
54:4TAG (18:1/18:1/18:2)	0.93	0.919	0.97	0.950	0.37	0.038*
56:6TAG (18:0/18:2/20:4)	2.00	0.209	2.75	0.016*	0.82	0.500
58:10TAG (16:0/20:4/22:6)	1.40	0.311	2.07	0.020*	0.88	0.509
58:11TAG	2.44	0.038*	1.92	0.027*	1.70	0.205
58:11TAG	1.87	0.030*	1.69	0.176	1.64	0.383
58:12TAG (18:3/20:4/20:5)	2.06	0.032*	1.91	0.168	2.65	0.005**
60:10TAG	1.68	0.131	2.24	0.021*	0.90	0.762
60:11TAG (16:2/22:4/22:5)	1.43	0.178	2.04	<0.001***	1.51	0.168
60:11TAG (16:0/22:5/22:6)	1.66	0.335	1.98	0.007**	1.39	0.401
60:11TAG (18:2/20:3/22:6)	1.80	0.018*	2.13	0.005**	1.42	0.304
60:12TAG (18:2/20:5/22:5)	1.67	0.106	1.75	0.177	2.80	0.011*
60:12TAG (18:3/20:4/22:5)	1.38	0.375	2.85	0.007**	1.37	0.356
60:12TAG (18:3/20:4/22:5)	2.25	0.039*	2.08	0.068	2.81	0.087
60:12TAG (18:3/20:4/22:5)	2.29	0.154	2.04	0.021*	1.64	0.308
60:13TAG (18:2/20:5/22:6)	1.66	0.086	2.00	0.015*	2.58	0.067
62:11TAG (18:2/22:4/22:5, 16:1/22:5/24:5)	1.64	0.063	1.77	0.031*	1.58	0.232
62:12TAG (18:3/22:4/22:5)	1.56	0.147	2.27	0.003**	1.35	0.370
62:13TAG (16:1/22:6/24:6, 20:2/20:5/22:6)	2.28	0.121	1.77	0.003**	1.82	0.363
62:13TAG (18:1/22:6/22:6)	2.09	0.106	2.36	0.029*	2.53	0.272
62:14TAG (18:2/22:6/22:6)	2.25	0.119	2.45	<0.001***	2.20	0.274
64:14TAG	1.57	0.215	2.06	0.008**	1.95	0.251
65:14TAG	1.83	0.052	2.03	<0.001***	1.52	0.299
***Cholesterol ester (ChE)***						
18:1ChE	0.74	0.016*	0.78	0.089	1.12	0.423
18:2ChE	1.16	0.170	1.17	0.029*	1.16	0.307
20:5ChE	1.38	0.041*	0.93	0.762	1.33	0.138

^1^ Fold changes (ratio of APP/tau to wild-type) are means of N=3-5.

^2^ Welch’s *t*-test (*p<0.05, **p<0.01,***p<0.001).

^3^ This specie was picked up not from OPLS-DA loading S-plot but manually.

**Table 4:** Fold changes of the significantly changed oxidized fatty acids in plasma

**Molecular species**	**4 months**		**10 months**		**15 months**	
	**Fold Changes **^**1**^	***p*-value **^**2**^	**Fold Changes **^**1**^	***p*-value **^**2**^	**Fold Changes **^**1**^	***p*-value **^**2**^
***Metabolites derived from arachidonic acid***						
17-HETE	1.59	0.018*	1.41	0.112	1.36	0.111
18-HETE	1.46	0.021*	1.48	0.105	1.67	0.011*
5,15-diHETE	2.02	0.002**	1.42	0.159	1.97	0.002**
***Metabolites derived from eicosapentaenoic acid***						
8-HEPE	1.55	0.125	1.65	0.095	2.00	0.006**
15-HEPE	1.20	0.314	1.47	0.197	1.51	0.020*
17,18-EpETE	1.66	0.165	1.46	0.157	1.85	0.004**
14,15-diHETE	2.16	0.032*	1.80	0.051	2.21	0.122
17,18-diHETE	2.11	0.003**	1.51	0.116	2.22	0.004**
***Metabolites derived from docosahexaenoic acid***						
7-HDoHE	1.28	0.336	1.46	0.102	1.46	0.044*
10-HDoHE	1.62	0.047*	1.11	0.550	1.52	0.252
19,20-diHDoPE	1.67	0.010*	1.42	0.027*	1.80	0.033*
***Metabolites derived from linolenic acid***						
9-HOTrE(alpha)	1.64	0.202	2.53	0.023*	2.08	0.108
13-HOTrE(alpha)	1.50	0.147	1.87	0.041*	1.73	0.008**

^1^ Fold changes (ratio of APP/tau to wild-type) are means of N=3-5.

^2^ Welch’s *t*-test (*p<0.05, **p<0.01,***p<0.001).

HETE, hydroxyeicosatetraenoic acid; diHETE, dihydroxyeicosatetraenoic acid; HEPE, hydroxyeicosapentaenoic acid; EpETE, epoxyeicosatetraenoic acid; HDoHE, hydroxydocosahexaenoic acid; diHDoPE, dihydroxydocosapentaenoic acid; HOTrE, hydroxyoctadecatrienoic acid .

### Concomitant changes in brain tissues and plasma

**Figure 3:**
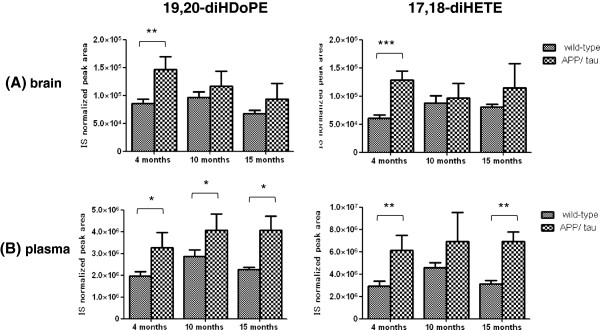
**Consistent changes in the levels of two ω-3 fatty acid metabolites in brain tissue and plasma.** The relative levels of 19,20-diHDoPE and 17,18-diHETE in brain tissue (A) and plasma (B) are represented by the IS normalized peak areas using leukotriene B_4_-d4 as an IS. *p < 0.05, **p< 0.01, ***p < 0.001 by Welch’s *t*-test. Values are mean ± SD of N = 3–5.

Finally, we assessed whether the significant changes in lipid species were consistent for brain tissues and plasma. We found that 34:2PC (16:0/18:2) was concomitantly increased in brains (1.63-fold, *p* = 0.014) and plasma (1.17-fold, *p* = 0.007) of APP/tau mice compared to wild-type mice at 10 months (Tables 1 and 3). Furthermore, 52:3TAG was decreased both in brain (0.4-fold, *p*=0.002) and plasma (0.58-fold, *p*=0.045) at 15 months (Tables 1 and 3). The levels of 19,20-dihydroxy-docosapentaenoic acid (19,20-diHDoPE) and 17,18-dihydroxy-eicosatetraenoic acid (17,18-diHETE) in APP/tau mice at 4 months were significantly higher both in brain (1.72-fold, *p* = 0.002 for 19,20-diHDoPE and 2.12-fold, *p* < 0.001 for 17,18-diHETE) and plasma (1.67-fold, *p* = 0.010 for 19,20-diHDoPE and 2.11-fold, *p* = 0.003 for 17,18-diHETE) than those from wild-type mice (Figure 3).

## Discussion

In the present study we performed a lipidomic analysis of brain tissue and plasma from APP/tau mice and corresponding control (wild-type) mice at both presymptomatic and post-symptomatic phases using RPLC-ESI-TOFMS, RPLC-ESI-linear ion trap-MS, and RPLC-ESI-triple quadrupole MS/MS. AD brains display several characteristic pathological features, including accumulation of amyloid plaques composed of Aβ and neurofibrillary tangles composed of hyperphosphorylated tau protein. APP/tau mice are suitable animal models to study lipid dysregulation over very early to late stages of AD because these animals are destined to develop the disease based on currently proposed pathogenesis mechanisms.

In brain tissue from our model mice, 22:6ChE levels were significantly increased in APP/tau mice compared to controls at all stages examined. In agreement with our results, 22:6ChE was reported to be increased in forebrains of mutated APP/presenilin-1 mice (11-fold, p < 0.01) and mutated APP mice (3-fold, borderline significance), but not of presenilin-1 mice (0.9-fold) at 9–11.5 months of ages, compared with wild-type mice [9]. In postmortem entorhinal cortex of AD patients, increased levels of ChE (1.7-fold, p = 0.027) were also observed, although the predominantly increased species was different between AD humans (16:1ChE, 16:0ChE and 18:1ChE) and mice with mutated APP (22:6ChE) [9]. The elevation in ChE levels in our mouse model had already occurred at 4 months of age (Figure 1) when Aβ accumulation was not yet observed (Additional file 1: Figure S1), which supports the idea that intracellular ChE levels regulate Aβ generation [15].

Other notable lipid dysregulation observed in brains of APP/tau mice was the reduced levels of several pPE species, particularly at the late stage of AD (15 months). In brains from AD patients, plasmalogen levels were reported to be decreased [16,17], suggesting that the mechanisms for this reduction could be common between humans and APP/tau mice. Marked increases in Aβ at 15 months of age might promote the formation of reactive oxygen species, which oxidize pPE, resulting in reduced pPE levels. It was reported that pPE decreases γ-secretase activities [18], and that Aβ destabilized the rate-limiting enzyme of plasmalogen synthesis, alkyl-dihydroxyacetonephosphate synthase, through peroxisomal dysfunction [19]. The levels of 38:2pPE (p18:1/20:1) were consistently decreased across the tested ages in APP/tau mice, although the pathophysiological significance of this lipid species in AD progression awaits evaluation. The elevated levels of 12-HETE observed in APP/tau mice at early stages of AD (10 months, Table 2) were also consistent with a previous report showing that brains of AD patients exhibited higher levels of 12-HETE than control subjects [20]. As suggested in human cases, 12-HETE may be linked to oxidative stress and neurodegeneration in AD as well as being involved in learning and memory processes [21].

The decreased levels of SM species with medium-chain fatty acids at late stages of AD were also in agreement with a previous study using human postmortem AD brains in which medium-length SMs such as d18:0/N20:0 and d18:1/N20:0 were shown to be slightly down-regulated (*p* < 0.05) [9]. These decreases might reflect demyelination as in the case of human AD brains described by Svennerholm and Gottfries [22]. Aβ42 was reported to accelerate SM degradation by activating neutral sphingomyelinase [23].

Increased levels of 60:12TAG bearing two DHAs (16:0/22:6/22:6, 1.33-fold) in 10 month old animals were in agreement with the previous report in which the levels of 56:7TAG containing docosahexaenoic acid (22:6FA) were increased in entorhinal cortices from AD patients. Since DHA-conjugated ChE was also increased in brains from APP/Tau mice at all ages tested (Figure 1), the AD brain appears to accumulate DHA in both TAG and ChE forms during the early symptomatic phase. These lipids might be involved in the production of DHA-derived messenger molecules such as neuroprotectin D1, which are reported to play neuroprotective roles in AD brain [8]. On the other hand, most APP/tau mice TAG levels were decreased compared with wild type mice at 15 months, particularly 52:3TAG (16:0/18:1/18:2, 16:1/18:1/18:1), 53:6TAG and 54:4TAG (16:0/18:0/20:4). A decrease in AA containing TAG (58:7TAG) was also observed in the prefrontal cortices of AD patients and in forebrains from 9–11.5 month old presenilin 1-APP mice compared to control animals [9]. Hydrolysis of these TAGs might thus be enhanced for their conversion into various structural and signaling lipid molecules, including eicosanoids, DAGs, monoacylglycerols (MAG) and phospholipids.

In addition to the changes described above, significant decreases in PCs and PEs mainly with PUFAs were also detected during the late symptomatic phase. These phenomena might reflect fatty acid chain oxidation caused by the increased oxidative stress derived from increased Aβ peptide levels [24]. Several arachidonic acid metabolites in the brain also showed different levels when comparing APP/tau mice with their controls. Levels of PGD_2_, which mediates neural damage by Aβ_42_[25], were lowered at 10 months (Figure 2), reflecting a possible protective reaction in the brain. Inflammation is characteristic of AD brain tissues as aggregated Aβ and phosphorylated tau proteins are associated with increased levels of inflammatory cytokines such as interleukin-6 and tumor necrosis factor-α [26]. While levels of some cyclooxygenase (COX)-mediated metabolites such as TXB_2_ and 12-HHT were also decreased, others (PGE_2_, 6-keto PGF_1α_ and PGF_2α_) were not (Additional file 6: Figure S4). Overall, these results suggest that not all COX pathways but instead several downstream pathways (such as PGD_2_ synthesis) might be impaired in AD brains during the early symptomatic phase. 15-HETE is known to be a ligand for peroxisome proliferator-activated receptor γ (PPARγ) [27] and might have anti-inflammatory activity in the AD brain. In this context, decreases in 15-HETE levels in the early phase of AD might promote lowered resistance to AD progression. We also observed decreases in both 11,12-epoxy-5,8,11-eicosatrienoic acid (11,12-EpETrE) and 14,15-EpETrE during the early symptomatic periods (Table 2). The roles of these decreases in AD progression should be evaluated in the future.

Because metabolites that are consistently changed in both brain tissues and plasma from APP/tau mice compared to controls could be candidate diagnostic biomarkers for AD, we examined changes in lipid species levels in plasma. In both brain and plasma, 34:2PC (16:0/18:2) and 52:3TAG were consistently increased and decreased, respectively, in APP/tau mice compared with wild-type mice only after disease onset (10 or 15 months). Regarding oxidative fatty acid metabolites, 19,20-diHDoPE and 17,18-diHETE were consistently increased in both brain tissues and plasma from APP/tau mice before symptoms appeared at 4 months (Tables 2 and 4, Figure 3), and these increases were essentially maintained at 10 and 15 months of age in plasma but not the brain. 19,20-diHDoPE and 17,18-diHETE are stable metabolites of DHA and EPA, respectively, that are mediated by cytochrome P450 (CYP) and soluble epoxide hydrolase (EH). In mouse brain, CYP2C29, CYP2C37, CYP2C38, and CYP2C40 were identified as arachidonic acid epoxygenases [28] and appear to be responsible for EPA and DHA epoxidation. Furthermore, soluble EH was reported to be expressed in various regions of mouse brain, including the cerebral cortex and hippocampus [29]. Levels of the intermediate metabolites, 19,20-epoxy-docosapentaenoic acid (19,20-EpDPE) and 17,18-epoxy-eicosatetraenoic acid (17,18-EpETE), which are generated by P450 epoxygenase in neuronal and glial cells, were below the quantification limit in brain (data not shown) and thus appeared to be quickly metabolized into their stable metabolites, 19,20-diHDoPE and 17,18-diHETE, respectively. Although the physiological functions of these metabolites in the central nervous system are unknown, 19,20-EpDPE and 17,18-EpETE were shown to act as vasodilators in cerebral arteries [30]. Since increased blood pressure is a risk factor for AD [31], these metabolites might counteract the vasoconstrictive effects of Aβ. Furthermore, 17,18-EpETE was reported to have anti-inflammatory properties mediated through PPARγ in lung tissues [32]. Therefore, 19,20-EpDPE and 17,18-EpETE in the brain might play a protective role against the onset of AD, but they are rapidly metabolized into their less active metabolites, 19,20-DiHDoPE and 17,18-diHETE. In this context, the expression of microsomal EH was reported to be significantly elevated in the hippocampus and associated cortex in AD patients [33]. However, the exact mechanisms leading to the consistent increases in these two compounds in brain and plasma remain unknown.

During the pre-symptomatic phase, docosahexaenoyl lysoPC (22:6LPC) and eicosapentaenoy-lysoPC (20:5LPC) in plasma were significantly higher in APP/tau mice than in control mice (Table 3). LysoPC may represent a preferred physiological carrier of fatty acids to allow passage across the blood–brain barrier [34], and thus brain DHA and EPA might be at least partly supplemented by plasma 22:6LPC and 20:5LPC. The contribution of the lysoPC species to supplementation of DHA and EPA in the brain needs to be investigated.

A previous report showed that serum pPE levels were higher in Alzheimer’s disease patients than in age-matched controls [35] and increased Cer and decreased SM levels in plasma were observed in patients with early-stage AD [36]. However, these changes were not observed in our APP/tau mice (Table 3).

## Conclusions

We performed a lipidomic analysis using brain tissue and plasma obtained from APP/tau mice at three AD stages. Two lipid species, 38:2pPE (p18:1/20:1) and 22:6ChE, were consistently decreased and increased, respectively, across the tested ages in APP/tau mice compared to wild-type mice. As reported for human AD brains, brain tissue from APP/tau mice showed increased 12-HETE levels at the early symptomatic phase and decreased levels of both pPEs and SMs with medium chain fatty acids at the late symptomatic phase compared with control mice. In addition to these previously reported changes, 19,20-diHDoPE and 17,18-diHETE, the stable metabolites of 19,20-EpDPE and 17,18-EpETE that have a potential counteracting role for AD, were increased in APP/tau brain tissue at the pre-symptomatic phase, and concomitant increases in plasma were observed. Furthermore, 15-HETE, which has a potential protective role in AD, was decreased during the early symptomatic phase in APP/tau mice. Our results provide fundamental information on the further precise analysis of lipids in AD pathogenesis. These changes detected in APP/tau mice models of AD should be examined in patients in the near future.

## Abbreviations

AD: Alzheimer’s disease; Aβ: Amyloid β; APP: Amyloid precursor protein; BD: Bligh & Dyer; Cer: Ceramide; ChE: Cholesterol ester; CYP: Cytochrome P450; DAG: Diacylglycerol; DHA: Docosahexaenoic acid; 19,20-diHDoPE: 19,20-dihydroxy-4Z,7Z,10Z,13Z,16Z-docosapentaenoic acid; 17,18-diHETE: 17,18-dihydroxy-5Z,8Z,11Z,14Z-eicosatetraenoic acid; EH: Epoxide hydrolase; ELISA: Enzyme-linked immunosorbent assay; 19,20-EpDPE: 19,20-epoxy-4Z,7Z,10Z,13Z,16Z-docosapentaenoic acid; 17,18-EpETE: 17,18-epoxy-5Z,8Z,11Z,14Z-eicosatetraenoic acid; EPA: Eicosapentaenoic acid; ESI: Electrospray ionization; GM3: Ceramide-lactose-N-acetylneuraminic acid; HETE: Hydroxyeicosatetraenoic acid; MAG: Monoacylglycerols; PC: Phosphatidylcholine; PE: Phosphatidylethanolamine; pPE: Ethanolamine plasmalogen; PUFA: Polyunsatulated fatty acid; MF: Methyl formate; m/z: Mass-to-charge ratio; OPLS-DA: Orthogonal partial least-squares-discriminant analysis; PL: Phospholipid; PPAR: Proliferator-activated receptor; RPLC: Reversed-phase liquid chromatography; RT: Retention time; SM: Sphingomyelin; TAG: Triacylglycerol; TOFMS: Time of flight mass spectrometry.

## Competing interests

The authors declare that they have no competing interests.

## Authors’ contributions

Conception and design: MK, TR, NS, TO, SY. Execution of the experiments: TY, IM, MK, MM, SY, OA, MR. Data analysis and interpretation: MK, NMT, NH, IK, AM, TR, NS, TO, SY. Manuscript writing: TY, MK, NS, TO, SY. All authors have read and approved the manuscript and agree with submission to your journal.

## Supplementary Material

Additional file 1: Figure S1 Expression levels of Aβ_40_ and tau proteins in APP/tau mice. The levels of human Aβ_40_, and soluble (TBS-soluble plus sarkosyl-soluble) and sarkosyl-insoluble APP/tau mice at 4, 10, and 15 months of age were determined by ELISA as described in Materials and Methods. Values are mean ± SD of N = 3–5. Click here for file

Additional file 2: Figure S2 RPLC-ESI-TOFMS analysis of brain lipids from APP/tau and wild-type mice at 4 months of age. Total ion counts chromatograph (TIC) and two-dimensional map with retention time (RT) versus mass-charge (*m*/*z*) values of brain lipids measured by RPLC-ESI-TOFMS in the positive ion mode from wild-type (A) and APP/tau mice (B) at 4 months. The intensity of peaks is represented by color density spots. Lipid metabolites were eluted in the following order: lysophospholipids (Lyso PLs, LPLs) > phospholipids (PLs) = sphingomyelins (SMs) = ceramides (Cers) > triacylglycerols (TAGs) = cholesterol esters (ChEs), as indicated in the areas surrounded by the ellipses. Spots for ChEs were visible in APP/tau (B) but not wild-type mice (A). Click here for file

Additional file 3: Figure S3 Multivariate statistical analysis of brain lipids between APP/tau and wild-type mice at 4 months of age. OPLS-DA score plots and loading S-plots for brain tissues from APP/tau vs. wild-type mice at 4 months, derived from the RPLC-ESI-TOFMS data set (A: 0.1-37.5min in RT; B: 37.5-60min in RT). In score plots (wild-type is shown in open circle vs. APP/tau in closed circle), the goodness-of-fit parameter R2 and the predictive ability parameter Q2 were 1.000 and 0.840, respectively, for (A) and 0.897 and 0.619, respectively, for (B). Loading S-plot showed covariance w against correlation p (corr) of variables for discriminating components of OPLS-DA model. Cut-off values for w[1] > |0.05| and p (corr) > |0.6| were used to select metabolites that strongly contributed to the discrimination between APP/tau and wild-type mice, which are surrounded by the red dotted line. Click here for file

Additional file 4: Table S1 Relative quantification of lipid species meeting the OPLS-DA loading S-plot criteria in brain tissues. Click here for file

Additional file 5: Table S2 Relative quantification of the detected oxidized fatty acids in brain tissues). Click here for file

Additional file 6: Figure S4 Pathway analysis of arachidonic acid metabolites. HHT, hydroxy-heptadecatrienoic acid; HETE, hydroxyeicosatetraenoic acid; EpETrE, epoxyeicosatrienoic acid; TX, Thromboxane; COX, cyclooxygenase; LOX, lipoxygenase; CYP, cytochrome P450; PGFS, prostamide/prostaglandin F synthase; PTGIS, Prostacyclin synthase; PTGES, prostaglandin E synthase; PTGDS, prostaglandin D synthase; TBXAS, thromboxane-A synthase. Click here for file

Additional file 7: Table S3 Relative quantification of lipid species meeting the OPLS-DA loading S-plot criteria in plasma. Click here for file

Additional file 8: Table S4 Relative quantification of detected oxidized fatty acids in plasma. Click here for file
