# Supplementary material for: Lipidomic analysis of brain tissues and plasma in a mouse model expressing mutated human amyloid precursor protein/tau for Alzheimer’s disease
Source: Lipids Health Dis. 2013 May 9;12:68. doi: 10.1186/1476-511X-12-68 (PMC3668217; doi:10.1186/1476-511X-12-68)
Supplement: Additional file 1: Figure S1 — Expression levels of Aβ40 and tau proteins in APP/tau mice. The levels of human Aβ40, and soluble (TBS-soluble plus sarkosyl-soluble) and sarkosyl-insoluble APP/tau mice at 4, 10, and 15 months of age were determined by ELISA as described in Materials and Methods. Values are mean ± SD of N = 3–5. [file 1476-511X-12-68-S1.pptx]

## Slide 1
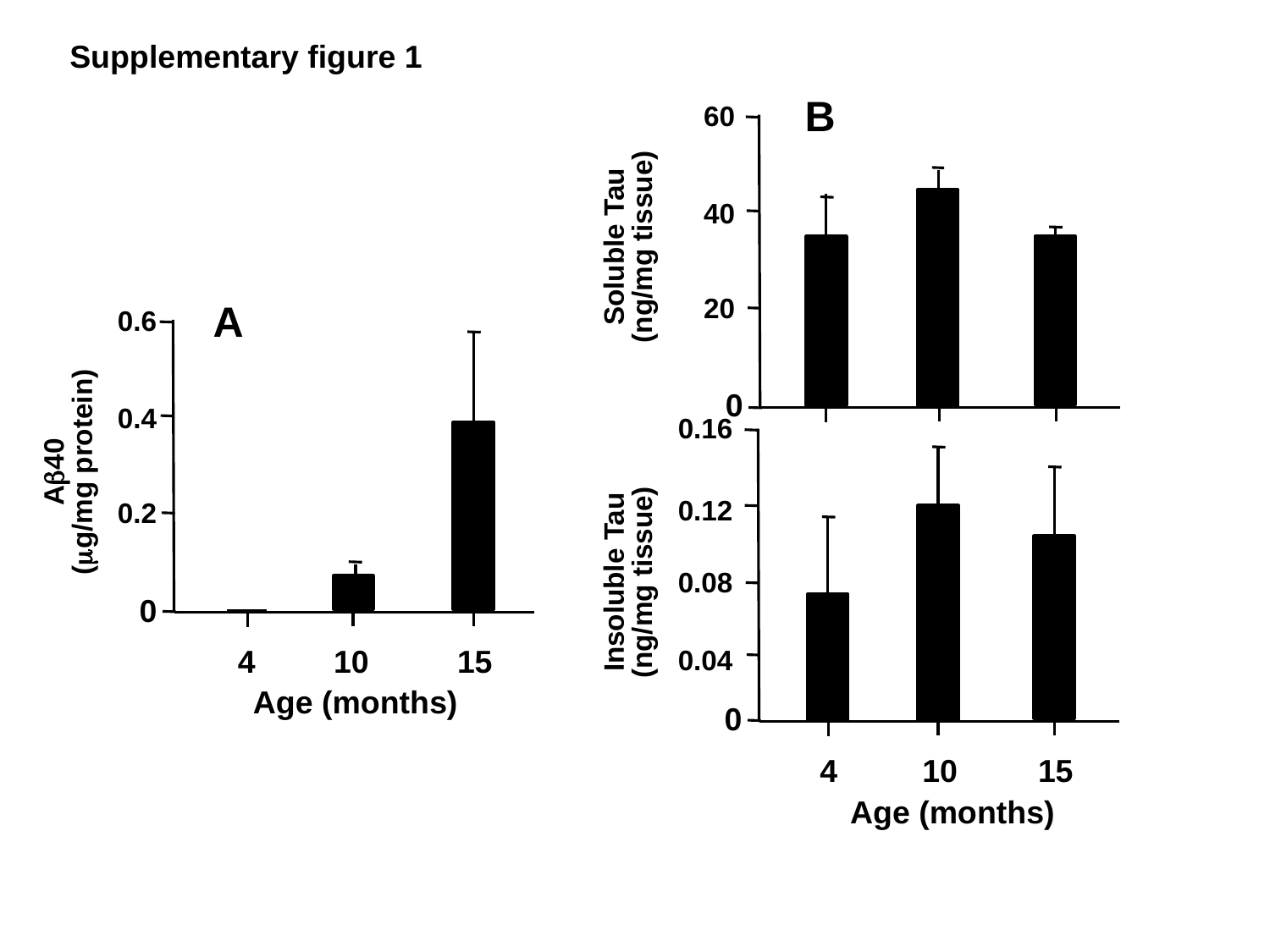

Supplementary figure 1
B
60
40
Soluble Tau
(ng/mg tissue)
20
A
0.6
0
0.4
0.16
Ab40
(mg/mg protein)
0.12
0.2
Insoluble Tau
(ng/mg tissue)
0.08
0
4
10
15
0.04
Age (months)
0
4
10
15
Age (months)
