# Supplementary material for: Lipidomic analysis of brain tissues and plasma in a mouse model expressing mutated human amyloid precursor protein/tau for Alzheimer’s disease
Source: Lipids Health Dis. 2013 May 9;12:68. doi: 10.1186/1476-511X-12-68 (PMC3668217; doi:10.1186/1476-511X-12-68)
Supplement: Additional file 2: Figure S2 — RPLC-ESI-TOFMS analysis of brain lipids from APP/tau and wild-type mice at 4 months of age. Total ion counts chromatograph (TIC) and two-dimensional map with retention time (RT) versus mass-charge (m/z) values of brain lipids measured by RPLC-ESI-TOFMS in the positive ion mode from wild-type (A) and APP/tau mice (B) at 4 months. The intensity of peaks is represented by color density spots. Lipid metabolites were eluted in the following order: lysophospholipids (Lyso PLs, LPLs) > phospholipids (PLs) = sphingomyelins (SMs) = ceramides (Cers) > triacylglycerols (TAGs) = cholesterol esters (ChEs), as indicated in the areas surrounded by the ellipses. Spots for ChEs were visible in APP/tau (B) but not wild-type mice (A). [file 1476-511X-12-68-S2.pptx]

## Slide 1
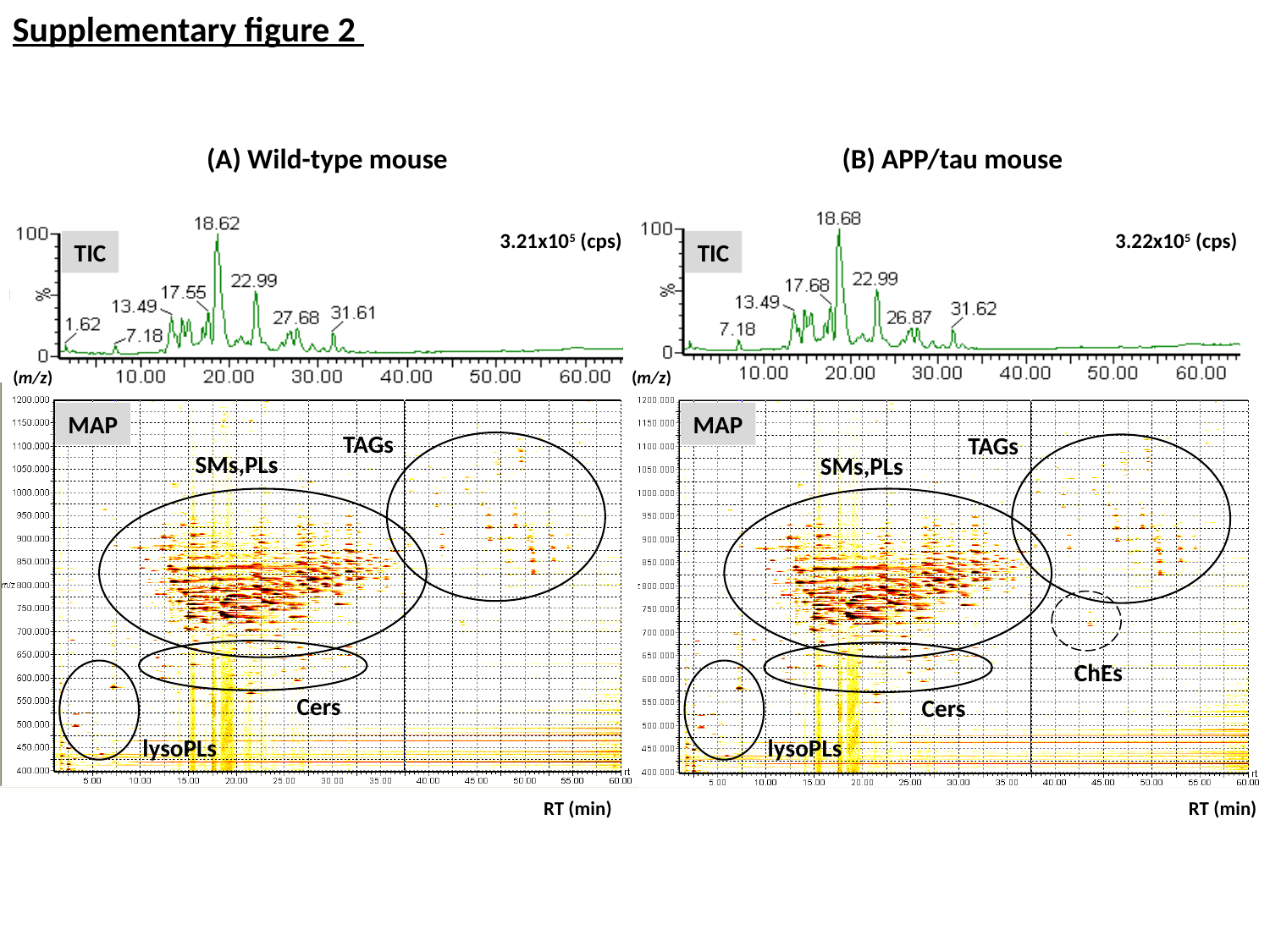

Supplementary figure 2
(A) Wild-type mouse
(B) APP/tau mouse
3.21x10^5^ (cps)
3.22x10^5^ (cps)
TIC
TIC
(m/z)
(m/z)
MAP
MAP
TAGs
TAGs
SMs,PLs
SMs,PLs
ChEs
Cers
Cers
lysoPLs
lysoPLs
RT (min)
RT (min)
