# Supplementary material for: Lipidomic analysis of brain tissues and plasma in a mouse model expressing mutated human amyloid precursor protein/tau for Alzheimer’s disease
Source: Lipids Health Dis. 2013 May 9;12:68. doi: 10.1186/1476-511X-12-68 (PMC3668217; doi:10.1186/1476-511X-12-68)
Supplement: Additional file 3: Figure S3 — Multivariate statistical analysis of brain lipids between APP/tau and wild-type mice at 4 months of age. OPLS-DA score plots and loading S-plots for brain tissues from APP/tau vs. wild-type mice at 4 months, derived from the RPLC-ESI-TOFMS data set (A: 0.1-37.5min in RT; B: 37.5-60min in RT). In score plots (wild-type is shown in open circle vs. APP/tau in closed circle), the goodness-of-fit parameter R2 and the predictive ability parameter Q2 were 1.000 and 0.840, respectively, for (A) and 0.897 and 0.619, respectively, for (B). Loading S-plot showed covariance w against correlation p (corr) of variables for discriminating components of OPLS-DA model. Cut-off values for w[1] > |0.05| and p (corr) > |0.6| were used to select metabolites that strongly contributed to the discrimination between APP/tau and wild-type mice, which are surrounded by the red dotted line. [file 1476-511X-12-68-S3.pptx]

## Slide 1
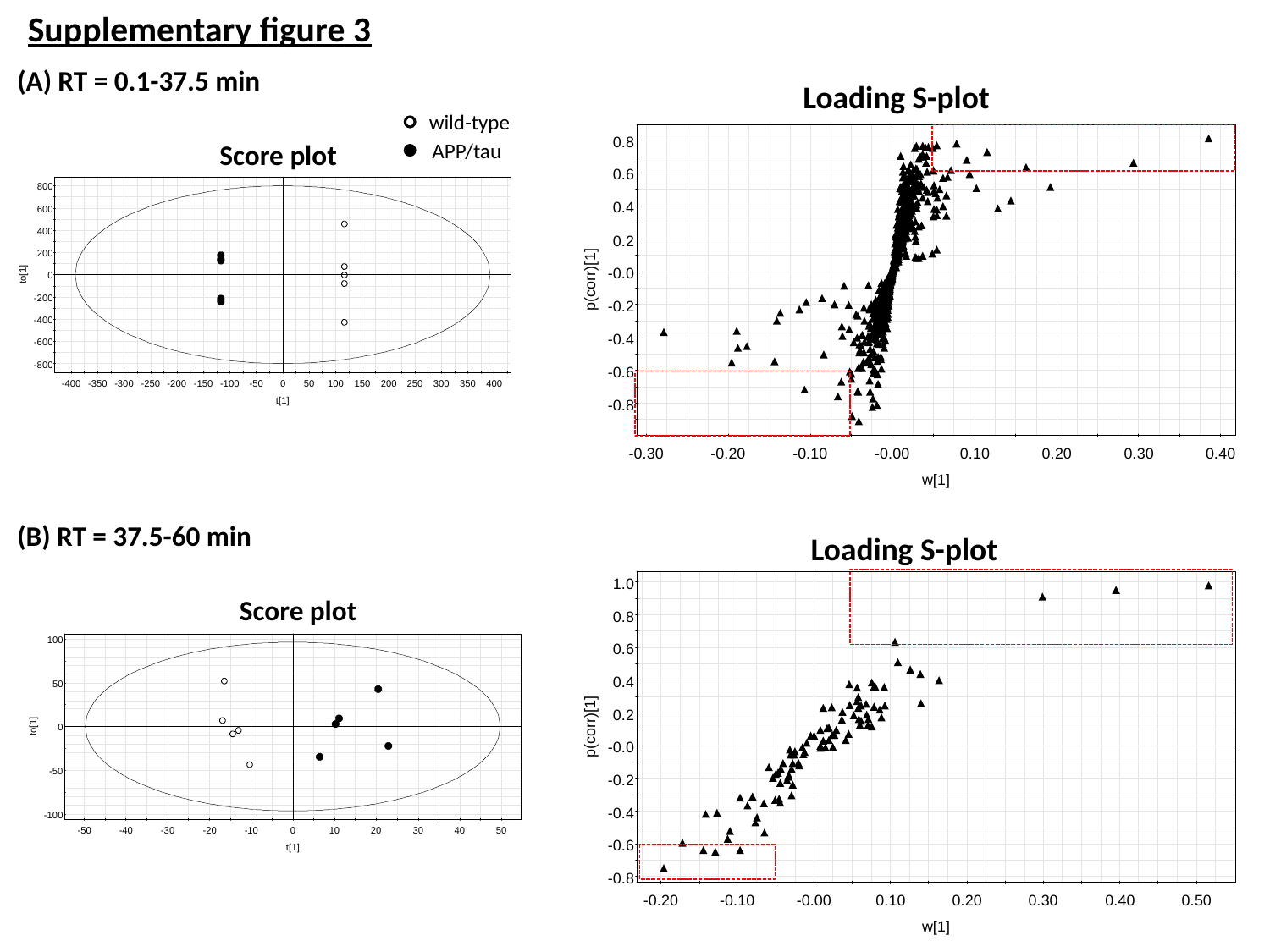

Supplementary figure 3
(A) RT = 0.1-37.5 min
Loading S-plot
wild-type
Score plot
APP/tau
(B) RT = 37.5-60 min
Loading S-plot
Score plot
