# Supplementary material for: Lipidomic analysis of brain tissues and plasma in a mouse model expressing mutated human amyloid precursor protein/tau for Alzheimer’s disease
Source: Lipids Health Dis. 2013 May 9;12:68. doi: 10.1186/1476-511X-12-68 (PMC3668217; doi:10.1186/1476-511X-12-68)
Supplement: Additional file 6: Figure S4 — Pathway analysis of arachidonic acid metabolites. HHT, hydroxy-heptadecatrienoic acid; HETE, hydroxyeicosatetraenoic acid; EpETrE, epoxyeicosatrienoic acid; TX, Thromboxane; COX, cyclooxygenase; LOX, lipoxygenase; CYP, cytochrome P450; PGFS, prostamide/prostaglandin F synthase; PTGIS, Prostacyclin synthase; PTGES, prostaglandin E synthase; PTGDS, prostaglandin D synthase; TBXAS, thromboxane-A synthase. [file 1476-511X-12-68-S6.pptx]

## Slide 1
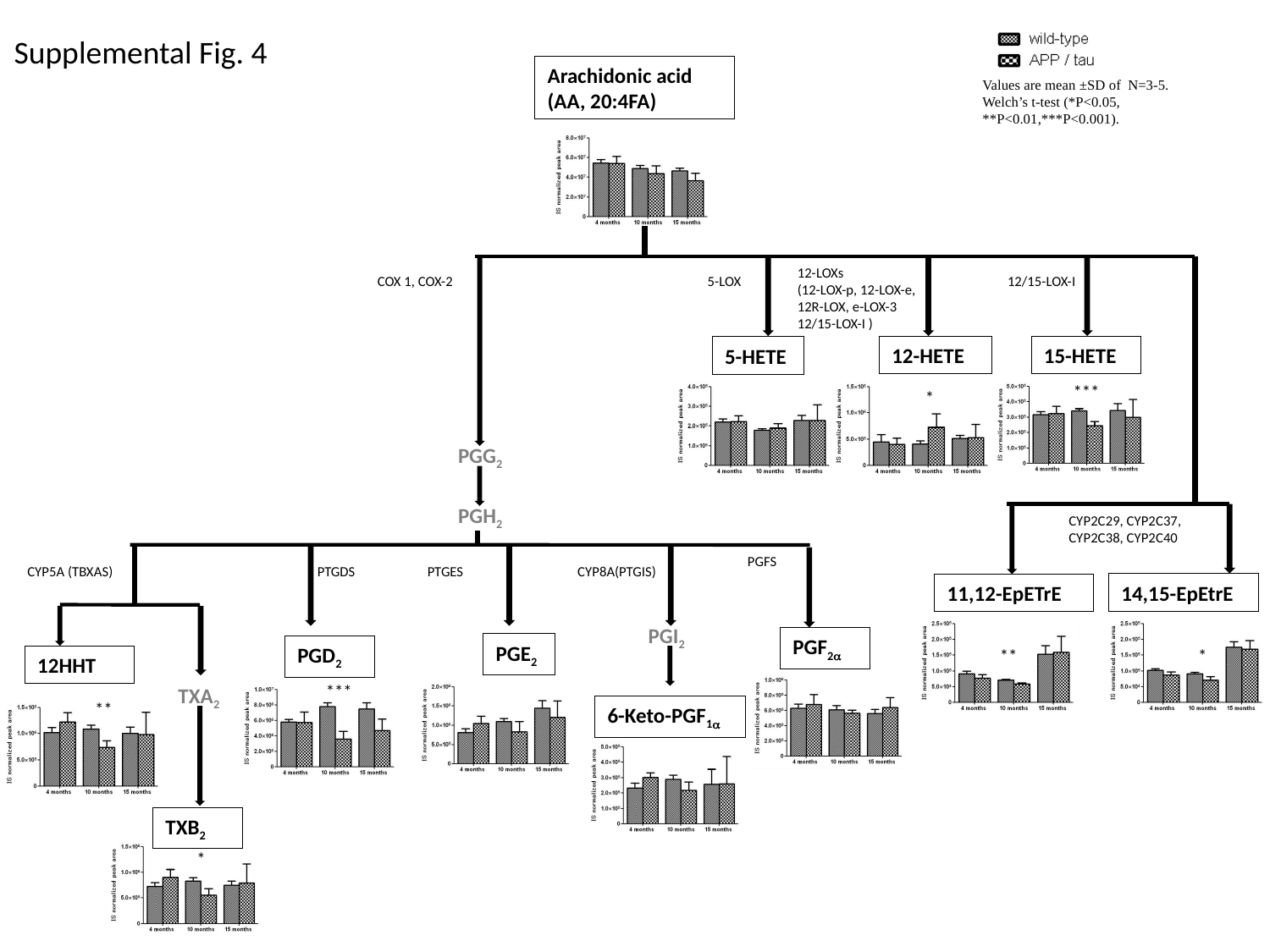

Supplemental Fig. 4
Arachidonic acid
(AA, 20:4FA)
Values are mean ±SD of N=3-5.
Welch’s t-test (*P<0.05, **P<0.01,***P<0.001).
12-LOXs
(12-LOX-p, 12-LOX-e, 12R-LOX, e-LOX-3
12/15-LOX-I )
COX 1, COX-2
5-LOX
12/15-LOX-I
12-HETE
15-HETE
5-HETE
***
*
PGG2
PGH2
CYP2C29, CYP2C37, CYP2C38, CYP2C40
PGFS
CYP5A (TBXAS)
PTGDS
PTGES
CYP8A(PTGIS)
14,15-EpEtrE
11,12-EpETrE
PGI2
PGF2a
PGE2
PGD2
**
*
12HHT
***
TXA2
**
6-Keto-PGF1a
TXB2
*
